# Supernumerary kidneys: a clinical and radiological analysis of nine cases

**DOI:** 10.1186/s12894-019-0522-0

**Published:** 2019-10-17

**Authors:** Peter Rehder, Rafael Rehwald, Julia M. Böhm, Astrid E. Grams, Alexander Loizides, Marco Pedrini, Jannik Stühmeier, Bernhard Glodny

**Affiliations:** 1 University Hospital for Urology, Department of Surgery, Medical University of Innsbruck, Innsbruck, Austria; 2 Department of Radiology, University of Cambridge, Cambridge, UK; 3 Queen Square Institute of Neurology, University College London, London, UK; 4 Department of Radiology, Medical University of Innsbruck, Anichstraße 35, 6020 Innsbruck, Austria; 5 Department of Neuroradiology, Medical University of Innsbruck, Innsbruck, Austria

**Keywords:** Supernumerary kidneys, Computed tomography, Renal variants, Diagnostic imaging, Duplex kidneys, Ureter fissus

## Abstract

**Background:**

A supernumerary kidney (SK) is an additional kidney with its own capsule and blood supply that is not fused with the ipsilateral kidney (IK). Because individual case reports indicate a high morbidity rate, the aim of this retrospective study was a detailed analysis of this rare anatomical variant.

**Methods:**

Our systematic imaging-based search for SKs, conducted in the period from 2000 and to 2017, yielded 9 cases in total (5 men, 4 women; mean age: 51.8 ± 22.8 years).

**Results:**

The SKs were observed on the right in six and on the left side in three cases. In six subjects (66%) they were located caudal and in three cases (33%) cranial to the ipsilateral kidney. Calculi were found in three (33%) of the renal collecting systems. Five (56%) of the SKs had hydronephrosis grade IV and one SK had recurrent pyelonephritis (11%). Two of the ureters opened into the ipsilateral seminal vesicle (22%). Two (22%) SKs were functional but atrophic. Clinically relevant findings were made in 33% of the IKs: atrophy (*n* = 2), calculi (*n* = 1), and reflux with recurrent pyelonephritis (*n* = 1); another 33% had anatomical anomalies without functional impairment. The correct diagnosis of a SK is possible using CT imaging in all subjects. The prevalence of SK based on CT imaging can be estimated to be 1:26750.

**Conclusions:**

CT is the method of choice for visualizing SKs. The correct diagnosis is crucial in preventing dispensable surgical procedures and for providing optimal patient treatment and outcome.

## Background

Supernumerary kidneys (SKs) are usually one [1], rarely two [2–5] additional kidneys, distinguished from the significantly more common duplex kidneys by the fact that they are not fused with the other kidney and have their own capsule and blood supply [1]. Most SKs are smaller than normal kidneys [6], but they can also be larger [1, 6, 7]. SKs are usually located caudal, less frequently cranial to the ipsilateral kidney [1]. While there is as yet no comprehensive description of the blood supply of SKs because of its high variability [1], the ureters of a SK tend to join with those of the ipsilateral kidney (IK) [1]. However, completely separate ureters draining into the bladder [1, 8], the vagina [9], the vulva [10], the prostatic urethra, or into vesical or vaginal pouches [1] have also been described.

Supernumerary kidneys have no inherent clinical relevance [1]. They are – however – often accompanied by urolithiasis, pyonephrosis, infections or hydronephrosis [1, 6, 11]. Individual cases have also been reported in which an associated adenocarcinoma [12], a Wilms tumor [13] or a cyst [7] were observed. Furthermore, SKs can be subject to traumatic injuries [14].

Formerly, most cases of SKs were detected during surgery or autopsy [6, 15]. Diagnostic methods such as retrograde pyelography [16], urography [12, 17–19], or angiography [12, 18–20] were less likely to lead to such a finding. Today, supernumerary kidneys are more frequently diagnosed using computed tomography (CT) [21], ultrasound, Tc-99^m^ MAG3 scintigraphy [22], Tc-99^m^ DTPA scintigraphy [23] or magnetic resonance imaging (MRI) [24].

One notable reason for the increased morbidity appears to arise from misinterpretations in the assessment of diagnostic procedures, in which SKs are not recognized as such. There may be no consequences [25], but this can at worst also lead to unnecessary interventions, such as biopsies [26] or surgery [27], including the risk of further complications [28].

The prevalence of SKs is unknown. There are only case reports of incidental findings of supernumerary kidneys, with a few exceptions of reports including two [1] or three cases [29, 30].

This study represents the yet largest case series, describing nine patients with supernumerary kidneys–the result of a systematic imaging-based assessment between 2000 and 2017. The aim of the present study was to estimate the frequency of SKs and to precisely describe and summarize both the anatomy and the clinical features of these patients for the first time.

## Methods

A SK was defined as organ with a typically asymmetric collecting system to which a ureter is connected, having its own capsule and blood supply and not being fused with the other ipsilateral kidney, distinguishing it from duplex kidneys [1]. The ureter of the SK may not open into the vagina in order to rule out confusion with Gartner’s duct cysts [31, 32], and no ureter may open into the presumed supernumerary kidney [32, 33]. The SK must be connected to the lower urinary tract [32, 34] and it must not be filled with blood or mucus at the onset of puberty [32, 35].

A total of 461,500 radiological examinations of the abdomen (without duplicate examinations), performed between 2000 and 2017 at our institution were systematically reviewed for anomalies of the kidneys: 214,000 contrast-enhanced CT scans acquired on spiral CT machines from different vendors (Genesis HiSpeed RP, QXi LightSpeed 4 Slice, LightSpeed 16, LightSpeed VCT 64, General Electric, W.I., USA; Somatom Sensation 4, 16 and 64 series, Somatom Definition Flash, Siemens AG, Erlangen, Germany), 31,500 MRI scans, performed on various systems (1.5 T Sonata, Symphony and Avanto, 3 T Magnetom Skyra, Siemens AG, Erlangen, Germany), 208,000 abdominal ultrasound examinations (HDI 5000, iU22, CX50 and EPIQ 7 ultrasound systems, Koninklijke Philips N.V., Best, the Netherlands; Xario 200, Aplio a550 and i800 series, Canon Medical Europe B.V., Zoetermeer, the Netherlands), as well as 8000 angiographies and urographies (Integris angiography unit, Koninklijke Philips N.V., Best, the Netherlands).

Most CT scans in patients with supernumerary kidneys were acquired after the intravenous application of iodinated contrast medium during a parenchymatous phase in 5 mm slice-thickness, while a urographic phase was almost always missing. All MRI examinations in which a supernumerary kidney was identified consisted of at least a T_2_-weighted sequence.

A significant part of the abdominal CT and MRI examinations was evaluated in the context of daily clinical routine. Additionally, the radiological reports of all included modalities were systematically screened for incidental findings of the urogenital tract, and the respectively identified imaging material carefully assessed. In a second step, every detected anomaly was independently re-evaluated and classified by both a senior board-certified radiologist (BG) and urologist (PR), resulting in nine cases of a SK beeing identified based on the inclusion criteria described (an organ with an asymmetric renal collecting system with an arising ureter, own capsule and blood supply and without fusion with the other kidney). A histopathological examination of the SK was available for four cases.

**Fig. 1:**
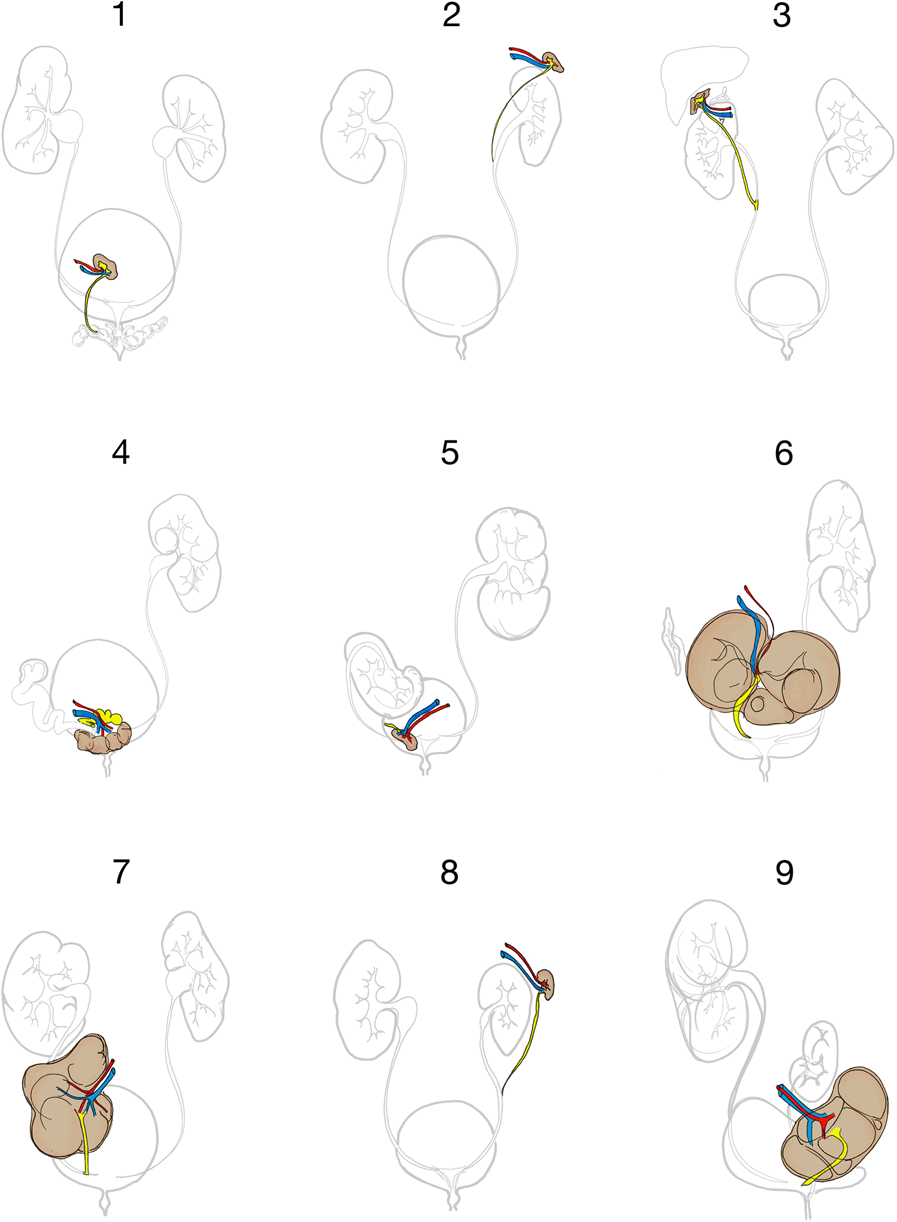
Illustration of the renal anatomy of the nine cases of supernumerary kidneys, derived from the abdominal imaging available for each individual patient. SK posterior to the bladder (Case 1; **a**); SK anterior to the upper pole of the ipsilateral kidney, sharing a common ureter (Case 2; **b**). SK cranial and lateral to the right kidney (Case 3; **c**; Fig. 2a and b). SK located posterior to the bladder (Case 4; **d**). SK cranial to the right seminal vesicle (Case 5; **e**). SK located posterior and cranial to the bladder, as an inferior crossed ectopia from left to right (Case 6; **f**). SK located caudal to the right kidney, sharing a ureter fissus (Case 7; **g**). SK was cranial to the left kidney, with a ureter fissus to the hydronephrotic IK (Case 8; **h**). SK located cranial and posterior to the bladder (Case 9; **i**)

**Fig. 2:**
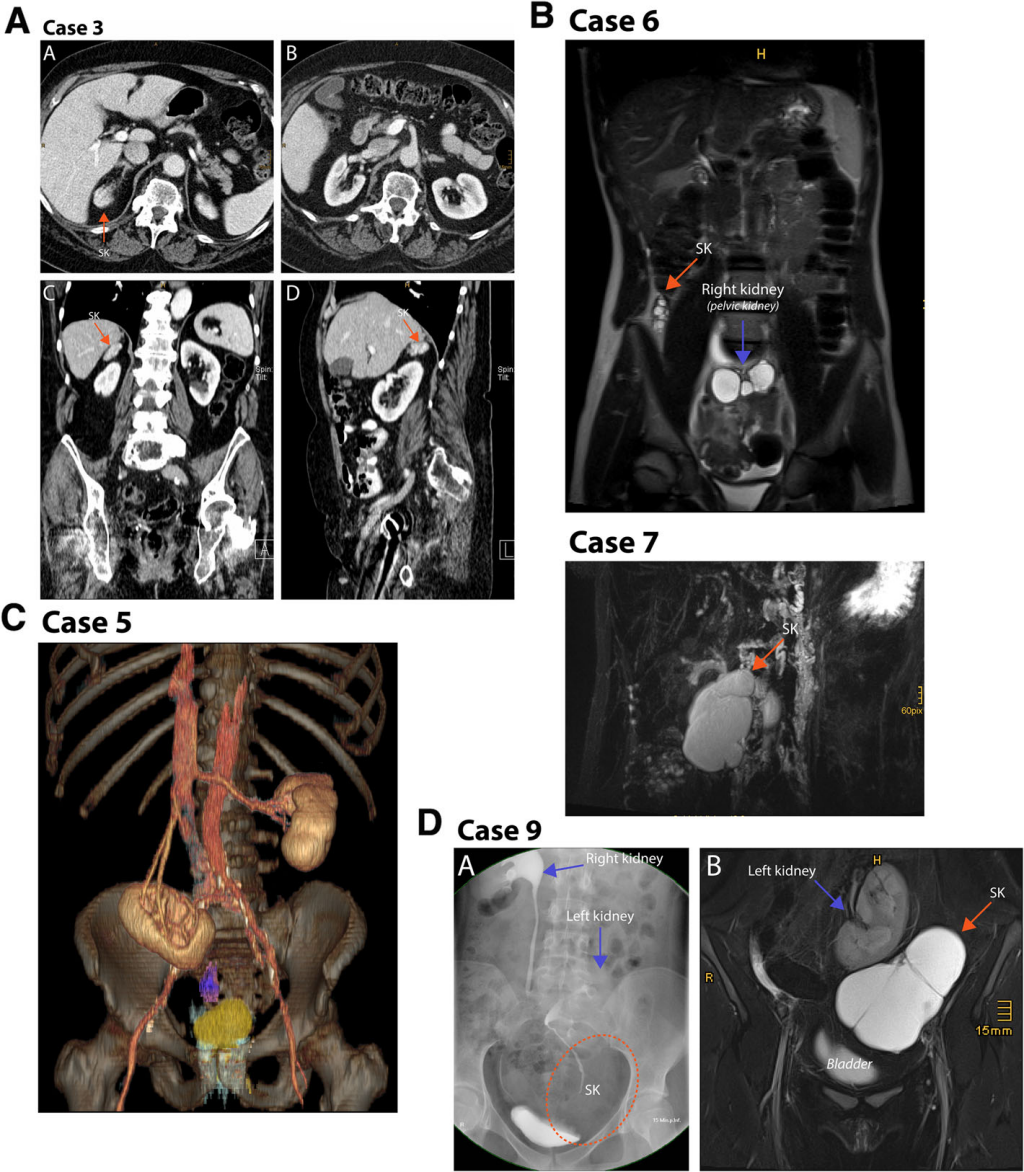
**Case 3**: (**a**) Transversal contrast-enhanced CT of a supernumerary kidney (SK) on the right, located dorso-lateral and at the same level as the ipsilateral adrenal gland. The SK presents with the same attenuation as the contralateral kidney. **b** Transversal contrast-enhanced CT of the normal kidneys bilaterally. **c** Sagittal CT-reconstruction. SK located cranial to the normal kidney. **d** Coronal CT-reconstruction with SK located cranial to the normal kidney. **Case 5:** Coronal 3D volume rendering reconstruction of a contrast-enhanced CT, SK in violet, bladder in yellow. **Case 6:** T_2_-weighted coronal image of the abdomen, showing the supernumerary kidney and the hydronephrotic right kidney (pelvic kidney). **Case 7:** T_2_-weighted thick-slab semi-coronal image of the supernumerary kidney and the normal kidney. **Case 9:** (**a**) T_2_-weighted coronal slice of the left kidney, the SK and bladder. (B) Intravenous pyelogram (15 min after i.v. contrast administration) depicting the right and left kidney as well as the supernumerary kidney as a mass and bladder

The renal anatomy of each identified SK has been comprehensively illustrated based on the imaging material available (Fig. 1), and additional coronal and sagittal CT and MRI reconstructions are presented for some patients in Fig. 2a-c, as these planes are considered particularly well suited to demonstrate supernumerary kidneys [36].

Descriptive statistics were performed using IBM SPSS Statistics for Windows, version 21 (IBM Corp., Armonk, N.Y., USA), with mean values and standard deviations given. For the statistical analysis, Fisher’s exact test was used for categorical and the Mann–Whitney U test for continuous variables. The sensitivity of the modalities was estimated under the assumption that kidneys can be reliably diagnosed using computed tomography [37]. The prevalence was determined based on the total number of patients examined between 2000 and 2017 with each imaging modality, respectively. A *p* < 0.05 was considered to be statistically significant.

## Results

Nine patients with a SK were identified; five men and four women (mean age 51.8 ± 22.8 years). The SKs were identified on the right in six cases and on the left in three cases (*p* = 0.132, Fisher’s exact test), caudal to the IK in six cases (cases 1, 4, 5, 6, 7, and 9) and cranial in three cases (cases 2, 3, and 8). The supernumerary kidneys are illustrated in Fig. 1a-I. Figure 2a-c shows sectional images, as well as ostensive images of selected cases.

Three out of nine patients (cases 4, 6, and 9; 33%) were clinically symptomatic due to the SK (infection and pain in case 4, pain in cases 6 and 9). The other cases were incidental findings (66%). None of the patients had initially had a urological examination; in three cases, urology was consulted later. The working diagnoses for the referral to imaging are listed in Table 1.

**Table 1:** Clinical details of the 9 patients with supernumerary kidneys

No.	Age group	Initial reason for radiological examination	Referred to radiology by?	Initial modality	Additional modalities	Correctly diagnosed	Initial diagnosis reported	Initial diagnosis to surgery	Patient management
**1**	Adult	Pneumonia and gastrointestinal bleeding	Pulmonology	CT	2 CT scans 1 Cystography	Yes	SK; diagnosis rejected 12 years later. DD: *“tumor”*	12 years	Watch-and-wait surgery 12 years later together with prostatectomy
**2**	Adult	Liver cirrhosis	Gastroenterology	CT	7 CT scans 1 MR scan	No	Described, but without diagnosis or interpretation	–	No consequence
**3**	Adult	Uterine Carcinoma	Gynecology	CT	3 CT scans 6 US exams	No	Metastasis of the adrenal gland	–	No consequence
**4**	Adult	Urinary tract infections	Surgery	CT	5 CT scans 4 US exam 2 Cystographies 3 MR scans	No	Abscess	2 days	Series of operations. Complications: urinary leakage; fistula to rectum and urine bladder
**5**	Adult	Lumbalgia	GP	CT	–	No	Recurrence of a presumably resected tumor	–	Watch-and-wait
**6**	Child	Lower abdominal pain	Pediatrics	US	1 CT 2 US exam 1 MR scan	Yes	SK	11 years	Resection of the hydronephrotic SK and ureter; shrunken IK remained in situ
**7**	Adult	Tumor palpated	Gastroenterology	CT	6 MR scans	No	Mesenterial cyst or lymphocele	–	No consequence
**8**	Adult	Aortic dissection	Surgery	CT	2 CT scans 1 US exam	No	Described, but without diagnosis or interpretation	–	No consequence
**9**	Adolescent	Inguinal hernia and pain	Surgery	US	2 US exams 1 Cystography 1 Urography 1 MR scan	No	Megaureter or cyst	17 years	Resection

Abbreviations: *DD* Differential diagnosis, *IK* Ipsilateral kidney, *SK* Supernumerary kidney, *UF* Ureter fissus, *US* Ultrasound

In two patients, the diagnosis had initially been made based on imaging (cases 1 and 6), in two other patients, the SKs were only described without further interpretation (cases 2 and 8). The other cases were misdiagnosed as a mesenteric cyst (case 7), a metastasis (case 3), an abscess (case 4), a megaureter (case 9) or a unknown tumor (case 5) (Table 2). Thus, the correct diagnosis had initially been made in only 22% of the cases, no diagnosis in 22% and an incorrect diagnosis in 66% of the cases.

**Table 2:** Anatomical details of the 9 patients with supernumerary kidneys

No.	Supernumerary kidney			Contralateral kidney	Ipsilateral kidney	Artery	Vein	Ureter	Complications		
	*Side*	*Size (cm)*	*Localization*						*Urologic*	*Genital*	*Other*
**1**	right	3.0 × 2.1 × 1.9	Dorsal of the bladder	Left, normal	Right, normal	Internal Iliac	Internal Iliac	Seminal vesicle	Concrements in SK without function	–	–
**2**	left	1.7 × 1.5 × 1.2	Ventral of the upper pole of the left kidney	Right, normal	Left, normal *(Ureter fissus)*	Aorta	Caval vein	Orifice in ureter of normal IK *(Ureter fissus)*	Concrements in SK	–	–
**3**	right	3.7 × 3.2 × 1.5	Cranial of the right kidney	Right, normal	Right, normal	Aorta	Caval vein	Orifice in ureter of normal IK *(Ureter fissus)*		–	–
**4**	right	8.5 × 8.1 × 6.5	Dorsal of the bladder, median and paramedian	Left, normal	Right; reflux; pyelonephritis; removed	Internal Iliac	Internal iliac	Bladder	Hydronephrosis IV in SK; Pyelonephritis in SK and IK	–	–
**5**	right	2.9 × 2.0 × 2.0	Dorsal of the bladder; cranial of the seminal vesicle	Left, normal	Right; pelvic kidney	Internal Iliac	Internal iliac	Seminal vesicle		–	6 lumbar vertebrae; Cleft of 6th vertebrate body arc
**6**	right	9.4 × 7.6 × 7.5	Dorsal and cranial of the bladder, median and paramedian	Left, normal	Right; pelvic kidney; cirrhosis of the kidney	Internal Iliac	Internal iliac	Bladder	Hydronephrosis IV in SK; cirrhosis of IK	–	Cleft of 5th vertebrate body arc
**7**	right	9.3 × 6.6 × 4.2	Caudal of the right kidney	Left, normal	Right, normal	Aorta	Caval vein	Orifice in ureter of IK *(Ureter fissus)*	Hydronephrosis IV in SK	–	–
**8**	left	6.7 × 5.2 × 6.2	Cranial of the left kidney	Right, normal	Cirrhosis of the kidney; kidney stones	Aorta	Caval vein	Orifice in ureter of IK *(Ureter fissus)*	Hydronephrosis IV in SK; cirrhosis of IK; kidney stones in common stem of ureter fissus	–	–
**9**	left	12.2 × 8.8 × 8.9	Caudal and lateral of the left kidney	Right, normal	Left; pelvic kidney	Internal Iliac	Internal Iliac	Bladder *(caudal)*	Hydronephrosis IV in SK; ipsilateral indirect inguinal hernia	–	Cleft of 5th vertebrate body arc

Abbreviations: *DD* Differential diagnosis, *IK* Ipsilateral kidney

The initial imaging modality was CT in seven subjects (78%) and ultrasound in the other two cases (cases 6 and 9; 22%). A total of 28 CT scans (8 patients; range: 1–8), 16 sonographies (6 patients; range 0–5), 12 MRI scans (5 patients; range 0–6), 4 voiding cystourethrograms (3 patients; range 1–2), 2 PET-CT scans (2 patients; range 0–1), 1 urography and one angiography (range 0–1) has been performed. Retrospectively, all initially acquired CT scans (100%), both PET-CT scans (100%), three of five initial MRIs (60%), and one of three voiding cystourethrographies (VCUG, 33%) were sufficient to establish the correct diagnosis. A sensitivity of 100% was achieved with CT and PET-CT, 60% with MRI and VCUG and 0% with angiography and sonography. Specificity estimates as well as positive and negative predictive values could not reliably be obtained, as discussed below.

Due to the number of individual examinations performed between 2000 and 2017, the period prevalence rates of SKs were estimated to be 1:26750 based on the CT scans and 1:31500 based on MR imaging. All other examinations were either performed for diagnostic purposes or were follow-up examinations after the initial CT scans.

None of the SKs were normal. The two (22%) still functional SKs (cases 3 and 5) were shrunken. Three of the collecting systems had calculi (cases 1, 2, and 8; 33%). Five SKs had hydronephrosis grade IV (cases 4, 6, 7, 8, and 9; 56%), and one of these suffered from recurrent pyelonephritis (case 4; 11%). Two ureters opened into the ipsilateral seminal vesicle (cases 1 and 5; 22%). The SK’s were with 5.7 ± 3.6 cm versus 9.2 ± 1.7 cm smaller than the IKs (*p* < 0.05).

Five patients underwent medical procedures related to their SK. One non-functioning SK with calculi which has been observed for 12 years was finally removed during a prostatectomy (case 1). In case 4, the patient presented externally with lower abdominal pain and fever. A hydronephrotic, orthotopic IK had been removed previously and the pyelonephritic SK in the small pelvis was misinterpreted to be an abscess. A vesicorectal fistula developed as a complication after the surgery. In case 5, imaging follow-up of the SK was planned, as a tumor was previously reported. The abdominal pain of patients 6 and 9 could be cured by surgical removal of their SKs.

Four out of the nine IKs were normal (cases 1, 2, 3, and 7; 44%). Three were ectopic and located caudally in the pelvis (cases 5, 6, and 9; 33%), one had calculi (case 8) and one had been removed because of recurrent pyelonephritis and vesicoureteral reflux (case 4). Four out of nine ipsilateral kidneys had a ureter fissus that branched to the SK (cases 2, 3, 7, and 8; 44%). Two of the kidneys were atrophic (cases 6 and 8, 22%). All contralateral kidneys were normal.

Three patients (33%) presented with a spina bifida occulta of the 5th (cases 6 and 9) or 6th lumbar vertebral arch (case 5). No other deformities were found.

## Discussion

The prevalence of supernumerary kidneys can be estimated to be 1:26750 with CT imaging. Supernumerary kidneys have been found more frequently located caudal than cranial to the ipsilateral kidney, and more often on the right than on the left side. All SKs were either hydronephrotic, atrophic, or had calculi while only two were still functional (22%). Three of the IKs (33%) were atrophic, hydronephrotic, or had calculi. However, only one third of the patients suffered from clinical symptoms; they all had pain that was cured after successful nephrectomy. The other cases can be considered as incidental radiological findings. Based on the criteria of a retroperitoneal organ with its own blood supply and capsule and an asymmetrical collecting system [1], all SKs could be identified as such using computed tomography as the imaging modality of choice.

This study has some limitations to consider. A histological examination of the SK was only available for four of the nine cases (cases 1, 4, 6, and 9). Furthermore, while the CT scans were of generally high quality, they were not specifically modified for optimal visualization of the kidneys. The retrospective estimate of the total number of examinations evaluated is also subject to a certain degree of uncertainty. One argument for the reliability is the good agreement of the prevalence of 1:27000 estimated based on CT and of 1:31500 based on MR imaging. The validity of the data regarding the accuracy of the presented diagnostic measures is however limited. Whereas the sensitivity of the different imaging modalities (100% for CT and PET-CT, 60% for MRI and VCUG, and 0% for angiography and sonography) can be considered reliable for at least in their magnitude, other markers of diagnostic accuracy–such as specificity, positive and negative predictive values as indicators of diagnostic effectiveness [38]–could not be obtained due to an unknown rate of true negative cases. Moreover, it has to be acknowledged that some of the STARD criteria have not been met by the present study [39]. The MRI and CT cohorts, for example, do at least partly differ and do not fully comprise of the same patients, whilst the readers of one modality were not blinded to the results and reports from other modalities. Finally, the number of patients is too limited to calculate confidence intervals.

While it was possible to make the diagnosis retrospectively in all of the CT scans, no supernumerary kidney was clearly identified by ultrasound, although the SKs were sonographically visualized in cases 6 and 9 and abdominal ultrasounds were available for 55% of the patients. Sonography thus appears to be not suitable for the reliable detection of SKs. In two cases in which MRI had been performed, the SK was not completely within the field of view. When fully scanned, it was possible to make the correct diagnosis. Only the PET-CT scan appears to allow the diagnosis to be made as reliably as CT imaging. It appears, that the main reason for an initially incorrect diagnosis in 78% of the cases is the relatively unknown nature of a SK which is not even considered as a differential diagnosis and that patients with unclear retroperitoneal masses are generally rarely referred to urology.

It can therefore be argued that there is currently no universally established diagnostic approach to reliably diagnose supernumerary kidneys. Performing an intravenous pyelogram or CT urography (CTU) assumes an intention to diagnose, something that has been missing in most, if not all, cases presented in this work. Furthermore, such a radiological approach would still most likely not solely be expedient to secure a definitive diagnosis, as no urinary excretion was observed from most of the SKs. A MRI scan of the kidneys acquired on an up-to-date system and utilizing a heavily T_2_-weighted sequence would allow for a higher detection rate and a more precise diagnosis. However, a justified suspicion before referring a patient to MRI is nevertheless both from a clinical and economic perspective advantageous. Additionally, the appropriate area of interest in which the kidney is suspected should be carefully selected, as even in modern scanners the field of view (FOV) is often highly focused.

In all cases, the renal artery and the renal vein could be clearly identified, likewise the ureter, and the abdominal organs were separate from the IK in all cases, so that the criteria of Geisinger [1] were fulfilled. Therefore, a differential diagnosis of a Gartner’s duct cyst combined with a Müllerian duct obstruction and ipsilateral renal dysgenesis [32] was not considered in any of the four female patients with SKs while, however, three such cases were identified but excluded.

While two of the SKs (cases 6 and 9) were larger than the other kidneys due to hydronephrosis grade IV, the other seven SKs were considerably smaller than regular kidneys. Only in cases 3 and 5 there no apparent cause other than atrophy as defined by Geisinger [1] is likely. Although the smallest supernumerary kidney was only 1.7 × 1.5 × 1.2 cm (case 2), it was not a so-called “*Beiniere*”, a term first introduced by Neckarsulmer [40] in 1914 to describe a kidney that was attached to the upper pole of the adjacent kidney like a hood [1]. We found no case of such a non-functional “*Beiniere*” [1] without an excretory duct [41] or with a rudimentary collecting system, but non-functioning parenchyma [40] that was connected with the IK [1]. Instead, in case 2, a narrow bridge to the adjacent IK was observed, as described by Geisinger [1] in his second case, but with the difference that the third kidney was located lateral, not medial to the upper pole of the IK. A functional SK of normal size thus likely to be rare.

While all patients had a normal contralateral kidney, four on the right and five on the left side, 66% of the IKs were conspicuous. An ureter fissus (cases 2, 3, 7, and 8), or caudal ectopia (cases 5, 6, and 9) are not clinically relevant. However, two IKs were shrunken, one of them with calculi, and a third had to be surgically removed because of recurrent pyelonephritis. Therefore, and as suggested by Rubin [42], special attention needs to be given also to the normal ipsilateral kidney.

Concomitant deformities occurred in 33% of the patients, consisting of fusion defects in the lumbosacral junctions which were clinically irrelevant and no further deformities were observed in other organ systems.

Detecting and correctly interpreting a supernumerary kidney by imaging is important for several reasons. Unnecessary procedures, as in case 1, could be prevented, and even more importantly complications of dispensable surgical procedures, such as in case 4, could be avoided, as the kidney should have been surgically removed *in toto*. The correct diagnosis is possible with a CT scan, and elaborate follow-ups would not be required. If there is no UF, the diagnosis can be confirmed by cystoscopy and verifying the presence of the ureter ostium. An accurate diagnosis is essential for providing an effective and successful therapy, which should ultimately consist of uretro-nephrectomy. This radical therapeutic approach is, however, only indicated if the kidney is clinically symptomatic. In conclusion, for the purpose of the most effective therapy, no generally valid treatment recommendations can be offered, except that each therapeutic decision should be tailored to the individual circumstances.

## Conclusion

In summary, our results show that SKs occur with a prevalence of 1:26750. None of the SKs were normal, however only one third of the patients had clinical manifestations. Surgery is rarely indicated, but can cure the pain that is sometimes present. Supernumerary kidneys can be identified by CT using Geisinger’s [1] criteria: a retroperitoneal organ with its own blood supply and own capsule and an asymmetric collecting system. In order to avoid unnecessary operations and the concomitant risk of complications, the correct diagnosis is of utmost importance for the individual patients.

## Data Availability

In accordance with the relevant statutory and legal provisions, the data as used in this publication are available from the corresponding author on reasonable individual written request. However, no information potentially identifying any subjects from this study will be shared.

## Abbreviations

CT: Computed tomography
CTU: Computed tomography urography
FOV: Field of view
IK: Ipsilateral kidney
MRI: Magnetic resonance imaging
PET: Positron emmision tomography
SK: Supernumerary kidney
UF: Ureter fissus
US: Ultrasound
VCUG: Voiding cystourethrography
